# Metabolomics Approach Based on Multivariate Techniques for Blood Transfusion Reactions

**DOI:** 10.1038/s41598-018-37468-9

**Published:** 2019-02-11

**Authors:** Seul Ji Lee, Haiping Wang, Soo Hyun Ahn, Mi Kwon Son, Gyu Hwan Hyun, Sang Jun Yoon, Jeongmi Lee, Jeong Hill Park, Johan Lim, Soon-Sun Hong, Sung Won Kwon

**Affiliations:** 1 College of Pharmacy, Seoul National University, Seoul, 08826 Korea; 2 Department of Mathematics, Ajou University, Suwon, 16499 Korea; 3 College of Medicine, Inha University, Incheon, 22212 Korea; 4 School of Pharmacy, Sungkyunkwan University, Suwon, 16419 Korea; 5 College of Pharmacy and Research Institute of Pharmaceutical Sciences, Seoul National University, Seoul, 08826 Korea; 6 Faculty of Pharmacy, Ton Duc Thang University, Ho Chi Minh City, Vietnam; 7 Department of Statistics, Seoul National University, Seoul, 08826 Korea

## Abstract

Blood transfusions temporarily improve the physical state of the patient but exert widespread effects on immune and non-immune systems. Perioperative allogeneic blood transfusions (ABT) are associated with various risks, including coagulopathy, incompatibility, transmission of infectious agents, and allergic reactions. Nevertheless, little is known about the global metabolic alterations that reflect the possible reactions of blood transfusions. In this study, we investigated metabolite changes generated by ABT in a rat model using metabolomics technology. To further profile the “metabolome” after blood transfusions, we used both liquid chromatography-quadrupole time-of-flight high-definition mass spectrometry and gas chromatography-mass spectrometry. ABT promoted a stimulatory microenvironment associated with a relative increase in glucose transporter 1/4 (GLUT1/GLUT4) expression. Supporting this result, glucose metabolism-related enzyme IRS1 and interleukin-6 (IL-6) were abnormally expressed, and levels of lysophosphatidylcholine (LysoPC) and its related enzyme phospholipase A2 (PLA2) were significantly altered in allogeneic groups compared to those in autologous groups. Finally, amino acid metabolism was also altered following ABT. Taken together, our results show a difference between autologous and allogeneic blood transfusions and demonstrate correlations with cancer-associated metabolic changes. Our data provide endogenous information for a better understanding of blood transfusion reactions.

## Introduction

As a lifesaving therapeutic treatment, there is a need for blood transfusions in patients undergoing surgery^1^ or with anemia^2^. However, blood transfusions still pose significant risks, including coagulopathy, incompatibility, transmission of infectious agents, and allergic reactions^3–7^. In a recent study, although blood transfusion reactions are rare, the risk of death, postoperative infection, and other adverse clinical outcomes was elevated among patients who received perioperative allogeneic blood transfusion (ABT)^8,9^. Moreover, based on the integration of data from observational studies via meta-analyses, significant associations between perioperative ABT and related cancer-specific mortality or cancer recurrence have been reported^10,11^. Accordingly, it is worth noting that the situations in which patients are perioperatively given ABT are likely to show side effects or induce cancer recurrence. Although a hypothesis regarding the genome and proteome, termed transfusion-related immunomodulation (TRIM), has been extensively proposed^12,13^, the molecules and mechanisms involved have not been fully elucidated^14^. Additionally, it is not known whether this problem is confined to immunosuppression.

Metabolomics involves the systematic study of endogenous metabolites and aims to comprehensively quantify and identify metabolites from biological samples that are the end products of cellular processes^15^. Gene expression data and proteomic analyses cannot provide a full description of the underlying physiology, and thus metabolomics is a useful supplement, offering a better understanding of physiological changes^16^. For the sake of gaining new insight into blood transfusions as well as to provide a new theoretical basis for clinical research, it is necessary to clarify the global metabolic alterations that accompany blood transfusions. The exploration of biomarkers contributes significantly to the development of supporting theoretical explanations for the results of clinical study. Distinguishing allogeneic blood transfusions from autologous blood transfusions may lead to the identification of critical biomarkers with adverse impacts on cancer patients who receive ABT for treatment^17^.

As a model for the study of humans, rats offer many advantages over mice and other organisms. More specifically, rats were once successfully used in blood transfusion research^18–20^. In this article, we established blood transfusion models in two strains of laboratory rats, Lewis rats and Sprague-Dawley (SD) rats, as all members of each strain are nearly genetically identical^21^. Moreover, due to the high sensitivity and selectivity of high performance liquid chromatography-quadrupole-time-of-flight combined with mass spectrometry (HPLC-Q-TOF-MS)^22^, it is often used to profile changes in endogenous metabolites. Additionally, gas chromatography combined with mass spectrometry (GC-MS) has particular advantages for the analysis of compounds with relatively low molecular weights^23,24^; therefore, more comprehensive metabolite profiling can be conducted by performing both HPLC-Q-TOF-MS and GC-MS.

This study focused on the differences between autologous and allogeneic blood transfusions in a rat model that provides a highly physiologically relevant setting for studying the interplay between blood transfusions and homeostasis in the microenvironment. The acquired data were optimized using a series of statistical approaches, and differential metabolites were identified using standards and databank-based MS/MS spectrum analysis. On the basis of the relevant literature and pathway databases, the biological natures of the various markers, including lipids, glucose, and amino acids, were discussed to further elucidate the possible mechanisms underlying the negative impact of ABT. We also found that levels of GLUT1/4, PLA2, IL-6, and IRS-1 varied in the plasma. Interestingly, these common transporters or signals differentially affected the regulatory cells involved in cancer metabolism. These findings suggest new non-clinical evidence of blood transfusion-associated impacts on cancer.

## Results

### Observation

Fourteen Lewis rats received a 1-mL transfusion of Lewis rat blood through the dorsal vein as a control (autologous) group, and 14 others received a 1-mL transfusion with SD rat blood as a test (allogeneic) group. Seven days after autologous blood transfusion and ABT, all of the experimental animals appeared in good condition, and no abnormalities were found. During the process of blood sampling, no hemolysis occurred.

### Global detection of biomarker candidates

We used HPLC-Q-TOF-MS and GC-MS to identify global differences in metabolites in rat blood following autologous and allogeneic blood transfusions. We implemented the empirical Bayes procedure by Efron^25^ [local false discovery rate (FDR)] to control for multiple testing errors that arise when testing many hypotheses simultaneously. At a local FDR level of 0.1, we tested both the null hypothesis (*H*_*p*0_) of no difference in the proportion of subjects with a non-zero intensity and the null hypothesis (*H*_*μ0*_) of no difference in the mean of the intensity. Therefore, we tested the overall null hypothesis $({H}_{0}={H}_{p0}{\cap }^{}{H}_{\mu 0})$ of no difference in both the proportion and mean of the intensity at a local FDR level of 0.2 (= 2 × 0.1).

More specifically, for the HPLC-Q-TOF-MS data, we considered the *i*^*th*^ metabolite as displaying a significantly different non-zero intensity proportion if ${p}_{pi}^{LC}\le 5.765\times {10}^{-3}$ at the local FDR level of 0.1. Also, at the local FDR level of 0.1, we considered the *i*^*th*^ metabolite as showing a significantly different mean of the intensity if ${p}_{\mu i}^{LC}\le 5.969\times {10}^{-7}$. As a result, we identified 474 significant metabolite features at a local FDR level of 0.2 based on the HPLC-Q-TOF-MS data. In the same manner, for the data from GC-MS, we identified 196 metabolite features satisfying ${p}_{pj}^{GC}\le 4.846\times {10}^{-2}$ or satisfying ${p}_{\mu j}^{GC}\le 6.812\times {10}^{-5}$ at a local FDR level of 0.2.

### Identification of differential metabolites

Identification of compounds detected by GC-MS was based on comparisons of mass spectra, retention indices (RIs), and authentic standards. According to comparison with the NIST and WILEY mass spectral databases registered in the GC-MS analysis system, followed by detection of the corresponding standards using GC-MS, 16 metabolites were identified. The combination of chromatographic properties and mass spectra gave an indication of a match to a specific compound (Fig. S4). In HPLC-Q-TOF-MS analysis, structural elucidation of metabolites should be routinely performed by the acquisition of additional MS data. Therefore, in the second step, we applied MS/MS experiments using an ion collision energy of 10–50 eV in the positive mode to obtain structural information via interpretation of the fragmentation patterns of the biomarker candidates. The typical mass error was less than 5 ppm. All lysophosphatidylcholines (LysoPCs) were confirmed based on characteristic fragments of 184.07, 104.11, and 86.1 m/z, as previously described^26,27^. These features were tentatively compared with those of the online database. The identification results confirmed that most of the candidates had a specific spectrum (Figs S5–S13). To qualitatively evaluate the confidence of metabolite identification, the standard LysoPC(17:0) was run on the same instrument with the same parameters.

Ultimately, 24 discriminant metabolites for distinguishing allogeneic from autologous blood transfusions were identified, including LysoPC (14:0), LysoPC (16:0), LysoPC (16:1), LysoPC (18:0), LysoPC (18:1), LysoPC (18:2), LysoPC (20:2), LysoPC (20:4), alanine, citric acid, glucose, glutamic acid, glutamine, glycine, isoleucine, lactic acid, lysine, ornithine, proline, pyroglutamic acid, serine, threonine, urea, and valine (Table 1).

**Table 1:** Identified metabolites that differentiate rats subjected to allogeneic (test) and autologous (control) blood transfusions.

	Potential biomarker	Molecular weight	−log_10_ (*p*)	Log_2_ (Fold change) (test/control)
GC-MS	Alanine	89.09	1.90	−12.49
	Citric acid	192.12	1.86	−3.56
	Glucose	180.16	6.33	1.89
	Glutamic acid	147.13	5.01	15.07
	Glutamine	146.15	19.79	−11.82
	Glycine	75.07	3.02	−2.02
	Isoleucine	131.18	11.32	−11.76
	Lactic acid	90.08	4.17	17.62
	Lysine	146.19	10.44	−15.32
	Ornthine	132.16	1.89	−10.52
	Proline	115.13	5.31	5.19
	Pyroglutamic acid	129.12	1.87	13.72
	Serine	105.09	2.64	2.99
	Threonine	119.12	6.14	17.49
	Urea	60.06	2.86	−13.52
	Valine	117.15	3.48	11.92
HPLC-Q-TOF-MS	LysoPC (14:0)	467.30	22.43	−2.32
	LysoPC (16:0)	495.33	2.56	−1.39
	LysoPC (16:1)	493.32	2.13	−0.94
	LysoPC (18:0)	523.36	3.38	−1.14
	LysoPC (18:1)	521.35	2.02	−1.29
	LysoPC (18:2)	519.33	2.32	−3.16
	LysoPC (20:2)	547.36	4.21	−1.23
	LysoPC (20:4)	543.68	3.47	−5.39

### Multivariate analysis

**Figure 1:**
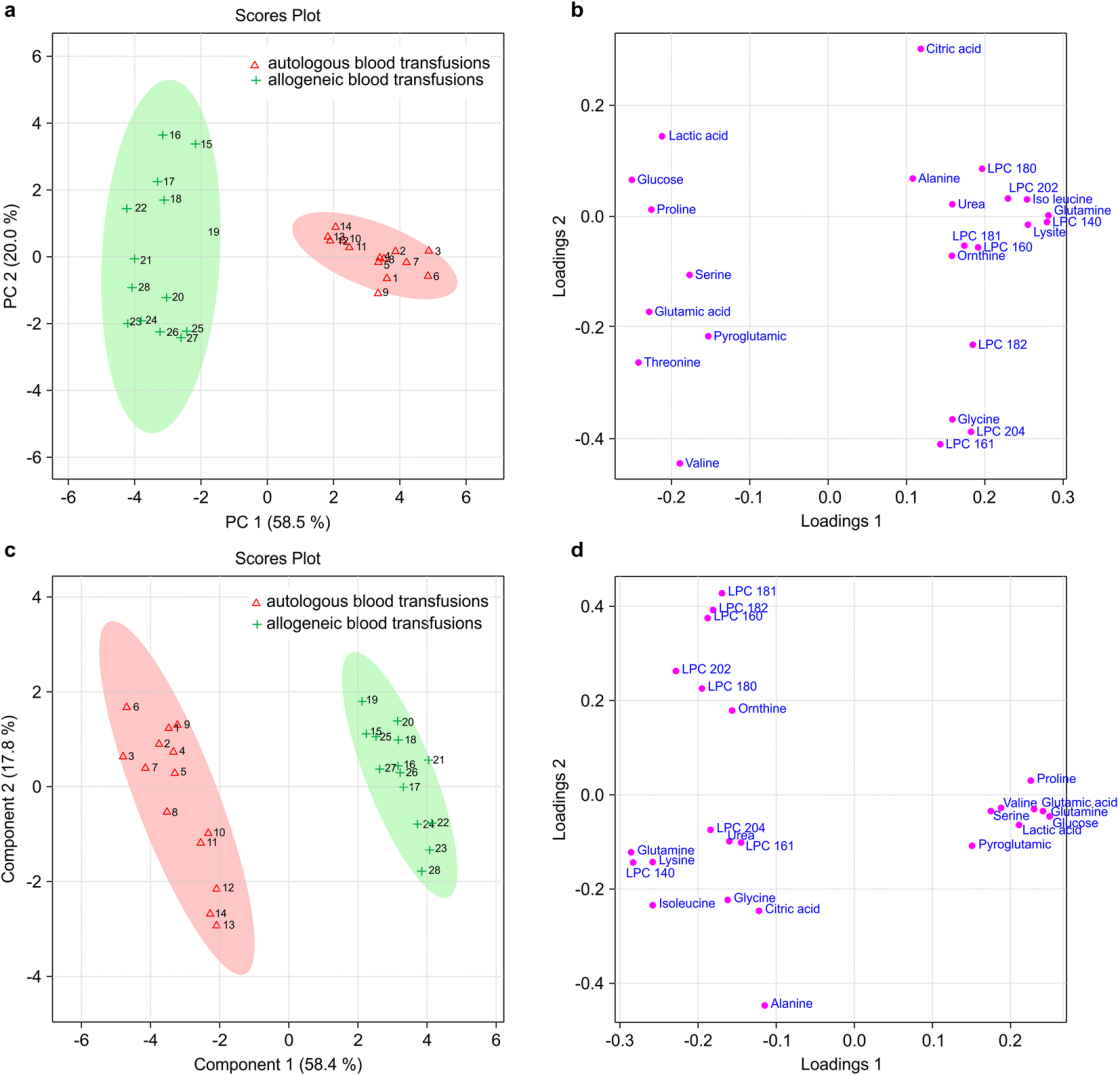
Multivariate statistical analysis based on non-targeted metabolite profile data derived from allogeneic (test) and autologous (control) blood transfusions. PCA (**a**) and PLS-DA (**c**) score plots for the first two components obtained from GC-MS and HPLC-Q-TOF-MS data. PCA (**b**) and PLS-DA (**d**) loading plots.

Principal component analysis (PCA) plots were generated to visualize the explanatory power of the identified marker candidates. The concentrations of the 24 marker candidate metabolites were confirmed to differ depending on the type of transfusion (allogeneic or autologous), as shown by the score plot, and the differences were confirmed by partial least squares discriminant analysis (PLS-DA) (Fig. 1).

**Figure 2:**
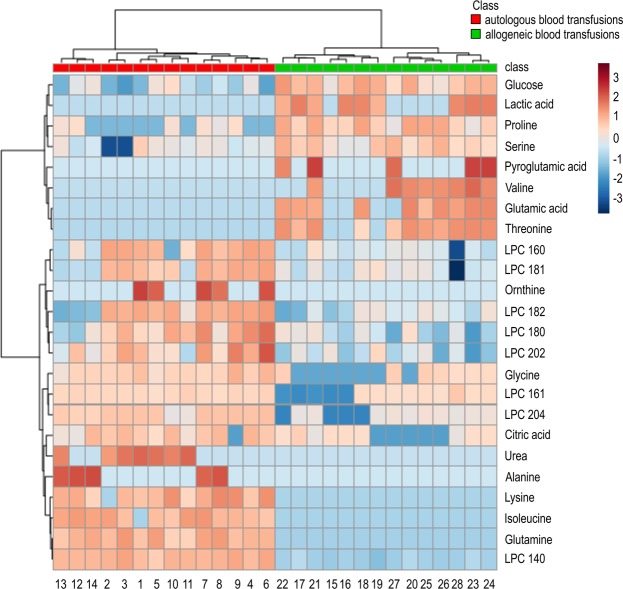
Heat map visualization of 24 significantly altered features in allogeneic (test) blood transfusion samples compared to those in autologous (control) blood transfusions. Shades of red and blue indicate increases and decreases, respectively, in the concentrations of metabolites. Clustering results are also shown. Euclidean distances were measured, and Ward’s clustering algorithm was used to construct the heat map.

Furthermore, clustering analysis was conducted using these 24 marker candidates. Samples from each of the blood transfusion types were shown to cluster as a group (Fig. 2), and a heat map of the clustering results was generated using Euclidian distances and Ward’s linkage.

### Sandwich enzyme-linked immunosorbent assay (ELISA)

**Figure 3:**
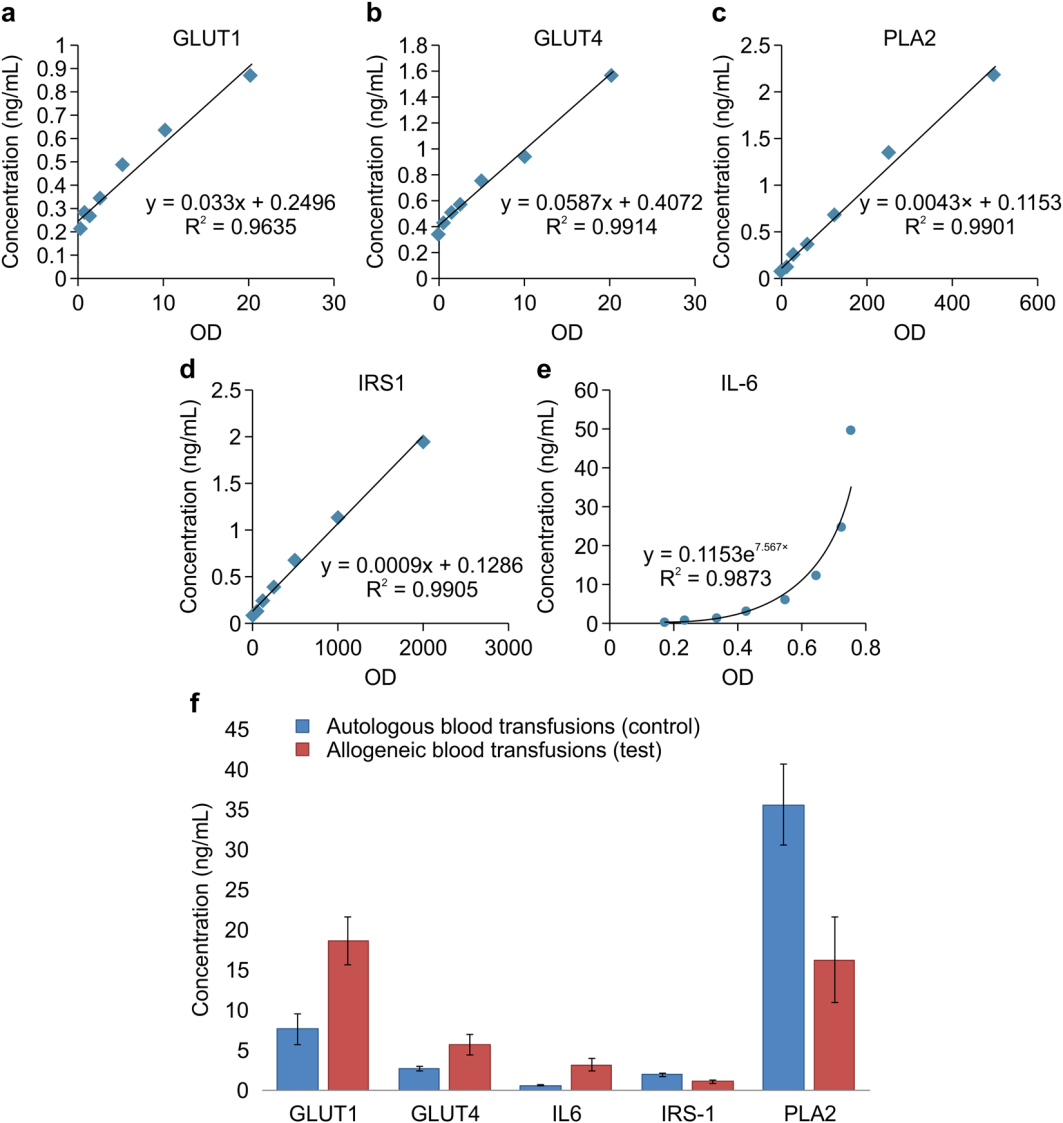
Calibration curves of the typical standards of GLUT1 (**a**), GLUT4 (**b**), PLA2 (**c**), IRS1 (**d**), and a rat IL-6 ELISA (**e**, exponential form) kit are shown. The calculated concentrations of the optical densities of GLUT1, GLUT4, PLA2, IRS1, and IL-6 (**f**) in sera from rats subject to autologous and allogeneic blood transfusions were determined by sandwich ELISA.

We analyzed IL-6, GLUT1, GLUT4, PLA2, and IRS1 levels in rat sera using sandwich ELISA, and each analysis included a standard curve. A representative standard curve for each of the five analyses is shown in Fig. 3(a–e). The linearity of each standard curve was confirmed, and R values of 0.99 (0.96 for GLUT1) were noted. The GLUT1, GLUT4, and IL-6 levels of the ABT test group were increased by approximately 143%, 116%, and 414%, respectively, compared with levels in the control group. In contrast, levels of PLA2 and IRS1 were approximately 54% and 44% lower than those of the autologous blood transfusion group, indicating the loss of activity of these two receptors (Fig. 3f). All five of these analyses showed significant differences (*p* < 0.05) between the autologous and allogeneic blood transfusion groups.

### Pathway analysis and interpretation

**Figure 4:**
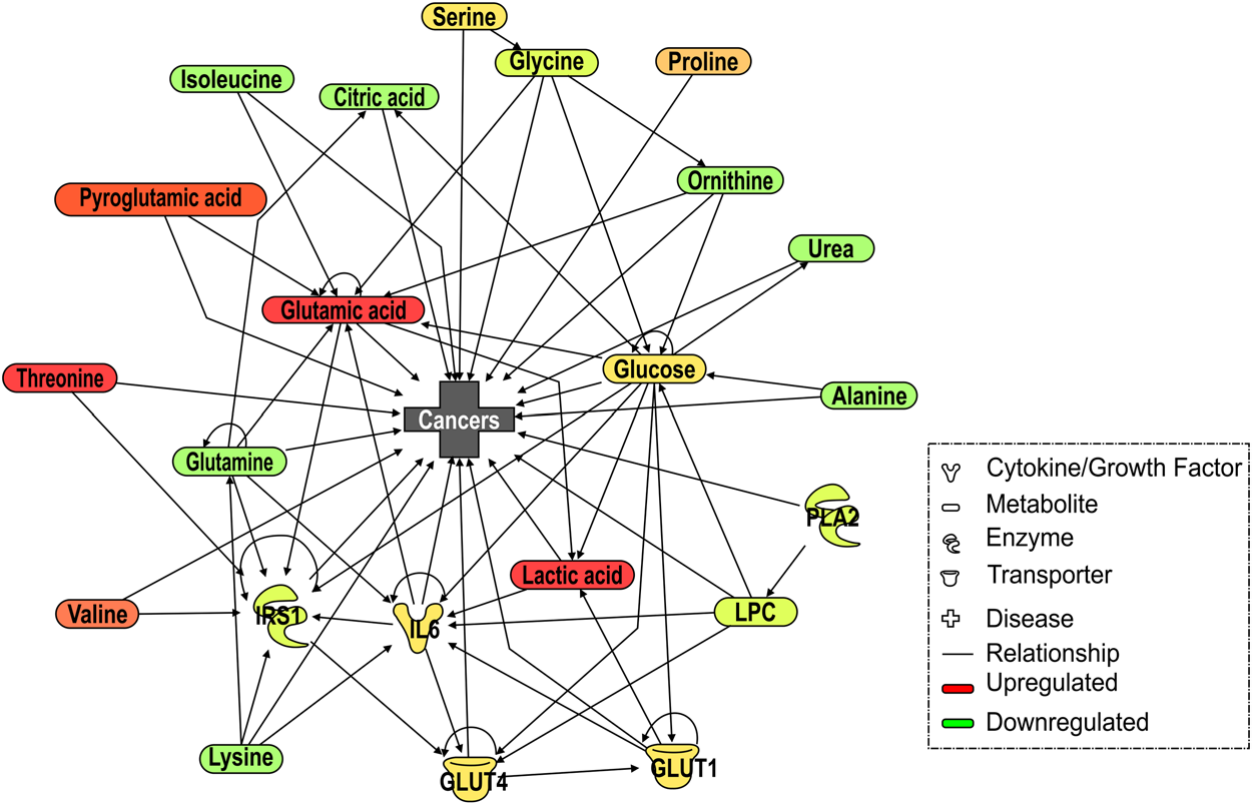
Metabolic pathway analysis of variations induced after ABT by IPA. (QIAGEN Inc.,https://www.qiagenbioinformatics.com/products/ingenuitypathway-analysis) Up- and downregulated metabolites and those with no change are shown in red, green, and yellow, respectively.

We next aimed to evaluate the obtained metabolomes in depth using Ingenuity Pathway Analysis (IPA), a web-based omics application for analyzing and interpreting metabolomes and transcriptomes. The results of pathway analysis showed that each metabolite or protein was directly or indirectly related to the others (Fig. 4). Notably, all 22 entities were found to be connected with the entity “cancer”, indicating that changes in endogenous expression as a result of ABT may be related to cancer. The results of the fold change analysis revealed that the metabolome expression of lysine, urea, alanine, glutamine, isoleucine, ornithine, LysoPC(20:4), citric acid, LysoPC(18:2), LysoPC(14:0), glycine, LysoPC(16:0), LysoPC(18:1), LysoPC(20:2), and LysoPC(18:0) in the allogeneic group was less than half that in the control group. Moreover, the metabolome expression of glucose, serine, proline, valine, pyroglutamic acid, glutamic acid, threonine, and lactic acid in the allogeneic group was more than twice that in the control group. Additionally, we used MetaboAnalyst to conduct an enrichment analysis of this pathway-associated metabolite set. The results indicated a significant Warburg effect (*p* = 0.00132, FDR = 0.0234) (Fig. S14).

## Discussion

In this study, we conducted for the first time a metabolic profile of rats undergoing allogeneic and autologous blood transfusions using GC-MS and HPLC-Q-TOF-MS to identify potential metabolic responses. Based on pathway analysis, the identified metabolites and proteins were found to be related to the entity “cancer”, implying a relationship between ABT and cancer.

The modulation of the immune systems of ABT recipients has been accepted as a condition known as TRIM^28^. As a key component of low-density lipoproteins (LDL)^29^, LysoPC regulates a variety of biological processes, including cell proliferation, tumor cell invasiveness, and inflammation. Not only does it promote inflammatory effects, including increased expression of endothelial cell adhesion molecules and growth factors, monocyte chemotaxis, and macrophage activation^30,31^, but it also is considered to be a potent regulator of T cell-activated inflammation at sites of tissue damage based on a previous study. Therefore, LysoPC is a natural adjuvant for the immune system, inducing humoral and cellular immune responses^32^. As a consequence, the reductions in LysoPC observed in the present study indicate that the suppression of T cells may occur in immune reactions after ABT, which may weaken the anti-tumor response. A similar phenomenon is described by the TRIM hypothesis, including the suppression of the activities of cytotoxic cells and monocytes, release of immunosuppressive prostaglandins, and increase in suppressor T-cell activity after blood transfusions^33–36^.

Another aspect of the metabolic regulation of the effector phase for tumor occurrence is the dependence on aerobic glycolysis in most cancer cells, a phenomenon termed “the Warburg effect.” Abundant glucose availability allows the production of ATP quickly through the aerobic glycolysis pathway, which also generates important metabolic intermediates for cancer cell growth and proliferation^37^. More recently, studies have demonstrated the high rate of glucose utilization by tumor cells, invoking the idea of using the Warburg effect against cancer cells by reducing glucose levels^38,39^. Our results show that in comparison to autologous blood transfusions, levels of glucose are higher after allogeneic blood transfusions, and this increase in glucose as a consequence of blood transfusion provides an excellent environment for the growth of cancer cells. Likewise, our results indicated that the expression of other glycolysis-related metabolites, such as citric acid, glutamine, glutamic acid, and lactic acid, was altered as well. We also conducted an enrichment analysis and showed that the marker candidates are associated with a Warburg effect. The activation of glucose transporters GLUT1 and GLUT4 together with glucose plays important roles in central carbon metabolism in cancer^40,41^. The potential connection between glucose growth and LysoPC and its relative receptors can be explained by the fact that LysoPC has been found to potentially enhance glucose uptake, reducing blood glucose levels in normal mice^42^. Additionally, in other studies, researchers have discovered a positive correlation between phospholipase A2 (PLA2) and glucose levels^43^. These results suggest that a reduction in LysoPC may directly or indirectly affect glucose homeostasis.

In addition, the results in this study indicate the up- and down-regulation of several amino acids following ABT, such as alanine, glycine, glutamine, isoleucine, lysine, proline, serine, threonine, and valine. Amino acid metabolism has been a focus of increased attention from cancer researchers and immunologists due to its importance in the metabolic reprogramming of proliferating cells. Many amino acid metabolic enzymes are described as immunosuppressive in the tumor microenvironment and represent targets for cancer therapy^44^. Therefore, it is expected that changes in the expression of these amino acids in the metabolome following ABT would have the potential to affect the development of cancer.

Based on the biomarkers identified in this study, we propose a metabolic pathway related to blood transfusion-induced changes. Our ELISA results help to illustrate the possible roles of different transporters and signaling molecules. The suppression of IRS1 and the activation of IL-6 are both reported to induce or promote tumor metastasis^45,46^. Although the pathways identified by previous studies^47–49^ indicated, in part, the dysregulation of LysoPC and glucose in some diseases, the elucidation of this pathway will facilitate our understanding of the likely effects of ABT. However, additional studies should be conducted to confirm the role of this pathway in blood transfusions and cancer recurrence.

## Methods

### Chemicals and reagents

All chemicals and reagents, unless otherwise stated, were purchased from Sigma-Aldrich Inc. (St. Louis, MO, USA). HPLC-grade chloroform, water, acetonitrile, and methanol were purchased from Avantor Performance Materials Inc. (Center Valley, PA, USA). The lipid standard was purchased from Avanti Polar Lipids (Alabaster, AL, USA).

### Rat model, blood transfusions, and sample collection

Twenty-eight male adult Lewis rats (160–180 g) were used as the transfusion recipients, while 14 adult Lewis rats and 14SD rats were used as blood donors. All animals were housed in individual cages and given food and water ad libitum throughout the study. Animals were observed for over two weeks prior to initiation of the study to check for evidence of pre-existing abnormalities.

Blood was obtained from donor animals by vena cava puncture and was transfused into recipients immediately after drawing to avoid the influence of anticoagulants and additional agents. Fourteen Lewis rats received a 1-mL transfusion^50^ with Lewis rat blood through the dorsal vein as a control group, and 14 others received a 1-mL transfusion with SD rat blood as a test group. This volume of donor blood was chosen to closely approximate the intravascular volume changes achieved in animals receiving 1 mL of whole blood^19^.

Animals were sacrificed 7 days after transfusion via cardiac air embolus. A total of 2 mL whole blood was obtained from each recipient by vena cava puncture through a laparotomy incision. Blood was placed in equal-volume heparin tubes before centrifugation at 1,300 × *g* and 4°C. Plasma was kept on ice for about 4 h before testing. The remaining samples were stored at −65°C until further analysis.

All rat experiments were approved and reviewed by Inha University (Approval ID: INHA 140321-283) and were performed in accordance with relevant guidelines and regulations.

### Sample preparation

#### GC-MS

To each 100 μL aliquot of plasma, 250 μL of solvent with a volume ratio of 2.5:1:1 of methanol, water, and chloroform was added, followed by vortexing for 1 min. After heating at 60°C for 30 min, the sample was centrifuged at 14,000 × *g* and 4°C for 5 min^51^. A 250-μL sample of supernatant was transferred to a clean test tube and dried under nitrogen at 20°C. The residue was then oximated with 40 μL methoxyamine hydrochloride (20 mg/mL in pyridine) and kept at 60°C for 60 min, followed by the addition of 20 μL *N*,*O*-bis(trimethylsilyl)trifluoroacetamide-trimethylchlorosilane (BSTFA-TMCS). The mixture was then kept at 60°C for another 45 min and filtered by centrifugation at 14,000 × *g* and 20°C for 10 min. The supernatant was collected for injection.

#### HPLC-Q-TOF-MS

Protein was precipitated with a threefold volume of acetonitrile (final concentration, 25%), followed by vortexing for 2 min and centrifugation at 14,000 × *g* and 4°C for 20 min. The supernatant was dried by purging with nitrogen. For analysis, samples were reconstituted in 100 μL acetonitrile/water (4:1) solvent^52^. Mixtures of pooled test group samples and pooled control group samples in a 1:1 (v:v) ratio with solvent were used as quality control (QC) samples.

### GC-MS analysis

GC-MS analysis was performed using a GCMS-QP2010 system (Shimadzu, Germany). Chromatographic separation was performed on a DB-5MS column (30 m × 0.25 mm, 0.25 μm, Agilent Technologies, Santa Clara, CA, USA). The GC oven temperature was held at 100°C for 5 min, increased to 180°C at a rate of 5°C/min, then increased to 300°C at a rate of 5°C/min and held at the final temperature for 5 min. A 1-μL sample was injected in the split mode, and helium (99.9999% He) was used as the carrier gas at a constant flow of 1 mL/min. The injection temperature and ion source temperature were 300°C and 200°C, respectively. After a 3-min solvent delay, mass spectra were obtained at 4 scans per second with a mass range of m/z 40–600. The ionization energy was 70 eV in the electron impact mode. Standards were injected for identification.

### HPLC-Q-TOF-MS analysis

LC-MS analysis was performed on an HPLC (Agilent) system equipped with an ODS column (100 × 2.1 mm, 1.7 μm) and coupled to an electron spray ionization quadrupole TOF-mass spectrometer (6510 ESI-Q-TOF-MS, maXis, Bruker, Billerica, MA, USA). The column temperature was 50°C with a flow rate of 0.35 mL/min. For non-targeted metabolomics, a linear gradient was applied for the first 26 min and then changed from 98% A (0.1% formic acid in water) to 100% B (acetonitrile) by holding for 20 min^52^. The mass spectrometer was operated in the ESI positive ionization mode, with ultra-high purity nitrogen as the nebulizer and drying gases (8.0 L/min) at a temperature of 200°C. The scan mode was applied for detection, and the scan mass ranged from 50 to 1000 m/z. For characterization and

QC data, a QC sample (a mixture of every sample) was injected at regular intervals during the run sequence. To obtain information for the identification of the metabolites, data-dependent MS/MS was performed by a collision energy ramp from 10 to 50 eV. All other parameters were the same as mentioned above. The standard LysoPC(17:0) was chosen to confirm the identification results. The entire process was performed using chromatography software (Bruker Daltonics).

### Data processing and statistical analysis

After data acquisition by HPLC-Q-TOF-MS and GC-MS, de-noising, baseline correction, and peak detection were performed. To exclude noise peaks in the subsequent analysis, MZmine 2.10^53^ (http://mzmine.github.io/) was employed for data pre-processing (Fig. S1).

We tested whether there was a significant difference in the intensity of each metabolite between control and treatment groups using R software. Noting that the observed data were derived from only a few samples and frequently had intensity values of zero for each metabolite, we compared not only the mean of the (log-transformed) intensity but also the proportion of detected samples (subjects having a non-zero intensity) for each metabolite between the two groups.

For the *i*^*th*^ or *j*^*th*^ metabolite, to compare the proportions of detected samples with a small sample size, we used a chi-squared test with continuity correction^54^ and observed the *p*-values ${p}_{pi}^{LC}$ based on the data from LC for *i* = 1, … 13872 and ${p}_{pj}^{GC}$ based on the data from GC for *j* = 1, …, 6089. Further, to test the mean difference, we considered an independent two-sample *t*-test using detected subjects and observed the *p*-values ${p}_{\mu i}^{LC}$ and ${p}_{\mu j}^{GC}$ based on the data from LC and GC, respectively.

### Identification of differential metabolites

Using GC-MS data, the selected metabolites were identified against standards, and a metabolite was deemed to be positively identified if the retention time and MS spectrum matched those of the authentic standards. For HPLC-Q-TOF-MS, MS/MS data were analyzed using a recently published strategy for the identification of selected metabolites^55,56^. Based on the accurate mass information, the Human Metabolome Database (HMDB) (http://www.hmdb.ca/), METLIN (http://metlin.scripps.edu/index.php), Massbank (www.massbank.jp/), Lipid Maps (http://www.lipidmaps.org/), and PubChem Compound (http://www.ncbi.nlm.nih.gov) were searched with a mass accuracy tolerance of 5 ppm to generate a list of mass-matched putative metabolites. MS/MS spectra emphasize neutral losses and product ions, which are characteristic of a metabolite group and allow for discriminating among database hits. The lipid standard LysoPC(17:0) was detected to verify the identification results by a comparison of its mass spectra and chromatographic retention time with those of plasma samples.

### Multivariate analysis

We analyzed the identified metabolites using MetaboAnalyst 4.0^57^ (http://www.metaboanalyst.ca/). Log transformation was performed to approximate a normal distribution. Data scaling was performed using the “auto scaling” function. As a result, a box plot and kernel density plot were obtained (Figs S2 and 3. The data were summarized by far fewer variables, called scores, which were weighted averages of the original variables. The weighted profiles were called “loadings.” PCA was conducted using the “prcomp” package. The calculation was based on singular value decomposition.

PLS regression is a control method for the extraction of data that predicts class membership (Y) from the linear combination of original variables (X) using multiple regression analysis techniques. A PLS regression was performed using the “plsr” function of the R “pls” package. Classification and cross-validation were performed using the corresponding wrapper function in the “caret” package. A permutation test was conducted to evaluate the importance of class distinction. In each permutation, a PLS-DA model between the data (X) and replaced class-label (Y) was constructed using the optimum number of components, which was identified from a cross-validation test against a model based on the original class assignment.

For the agglomerative hierarchical cluster analysis, each sample started in a separate cluster, and an algorithm combined them until all of the samples clustered together. A heat map was created as a visual aid in addition to phylogenetic trees. Hierarchical clustering was conducted using the “hclust” function in the “stat” package.

### ELISA

For analysis of IL-6 levels in rat sera, we used ELISA kits (R&D Systems, Minneapolis, MN, USA). The plates were coated with 100 μL of 2 μg/mL anti-IL-6 capture monoclonal antibody diluted in phosphate-buffered saline (PBS) for 24 h at room temperature. The plates were washed three times with PBS containing 0.1% Tween-20 and incubated with 100 μL/well of 1% bovine serum albumin (BSA, Sigma-Aldrich) in PBS for 1 h at room temperature. The rat sera and various concentrations of recombinant IL-6 were incubated overnight at 4°C. The plates were washed three times with PBS containing 0.1% Tween-20 and incubated with 100 μL/well of 50 ng/mL biotinylated anti-IL-6 detecting antibody for 2 h at room temperature. The plates were then washed and incubated for 30 min with 100 μL of horseradish peroxidase-conjugated streptavidin (Vector Laboratories, Burlingame, CA, USA). After washing, the 2,2-azino-bis substrate reaction was stopped by adding 50 μL of 2 N H_2_SO_4_. The absorbance was measured at 450 nm using a microplate reader. Ten rats were included in each group, and three replicate wells were used for each analysis. The levels of GLUT1, GLUT4, PLA2, and IRS1 in rat sera were also assessed using ELISA kits (Mybiosource, San Diego, CA, USA) according to the manufacturer’s instructions.

### Pathway analysis and interpretation

Data were analyzed through the use of IPA (QIAGEN Inc. https://www.qiagenbioinformatics.com/products/ingenuitypathway-analysis)^58^. After importing target metabolites and proteins as entities, we searched for diseases or phenotypes with as many connections as possible with the 22 entities. Concentration values were visualized, with shades of red, green, and yellow indicating up- and downregulation and no change, respectively, based on the log_2_ fold change in concentration between the allogeneic and autologous blood transfusion groups. We used MetaboAnalyst to conduct an enrichment analysis. Over-representation analysis (ORA) was implemented using the hypergeometric test to evaluate whether a particular metabolite set was represented more than expected by chance within the metabolite list; *p*-values were provided after adjusting for multiple testing.

## Supplementary information

supplementary information
