# Supplementary material for: Metabolomics Approach Based on Multivariate Techniques for Blood Transfusion Reactions
Source: Sci Rep. 2019 Feb 11;9:1740. doi: 10.1038/s41598-018-37468-9 (PMC6370787; doi:10.1038/s41598-018-37468-9)
Supplement: Supplementary file 1 — supplementary information [file 41598_2018_37468_MOESM1_ESM.docx]

Metabolomics Approach Based on Multivariate Techniques for Blood Transfusion Reactions

Seul Ji Lee^1^, Haiping Wang^1^, Soo Hyun Ahn^2^, Mi Kwon Son^3^, Gyu Hwan Hyun^1^, Sang Jun Yoon^1^, Jeongmi Lee^4^, Jeong Hill Park^5,6^, Johan Lim^7^, Soon-Sun Hong^3^, Sung Won Kwon^5,^*

^1^ College of Pharmacy, Seoul National University, Seoul, 08826, Korea

^2^ Department of Mathematics, Ajou University, Suwon, 16499, Korea

^3^ College of Medicine, Inha University, Incheon, 22212, Korea

^3^ School of Pharmacy, Sungkyunkwan University, Suwon, 16419, Korea

^5^ College of Pharmacy and Research Institute of Pharmaceutical Sciences, Seoul National University, Seoul, 08826, Korea

^6^ Faculty of Pharmacy, Ton Duc Thang University, Ho Chi Minh City, Vietnam

^7^ Department of Statistics, Seoul National University, Seoul, 08826, Korea

* To whom correspondence should be addressed:

Sung Won Kwon, College of Pharmacy, Seoul National University, Seoul, Korea, Tel.: +82-2-880-7844; Fax: +82-2-886-7844; E-mail: swkwon@snu.ac.kr

**Supplementary Figure 1.** **PCA plot of the entire detection results.**

**
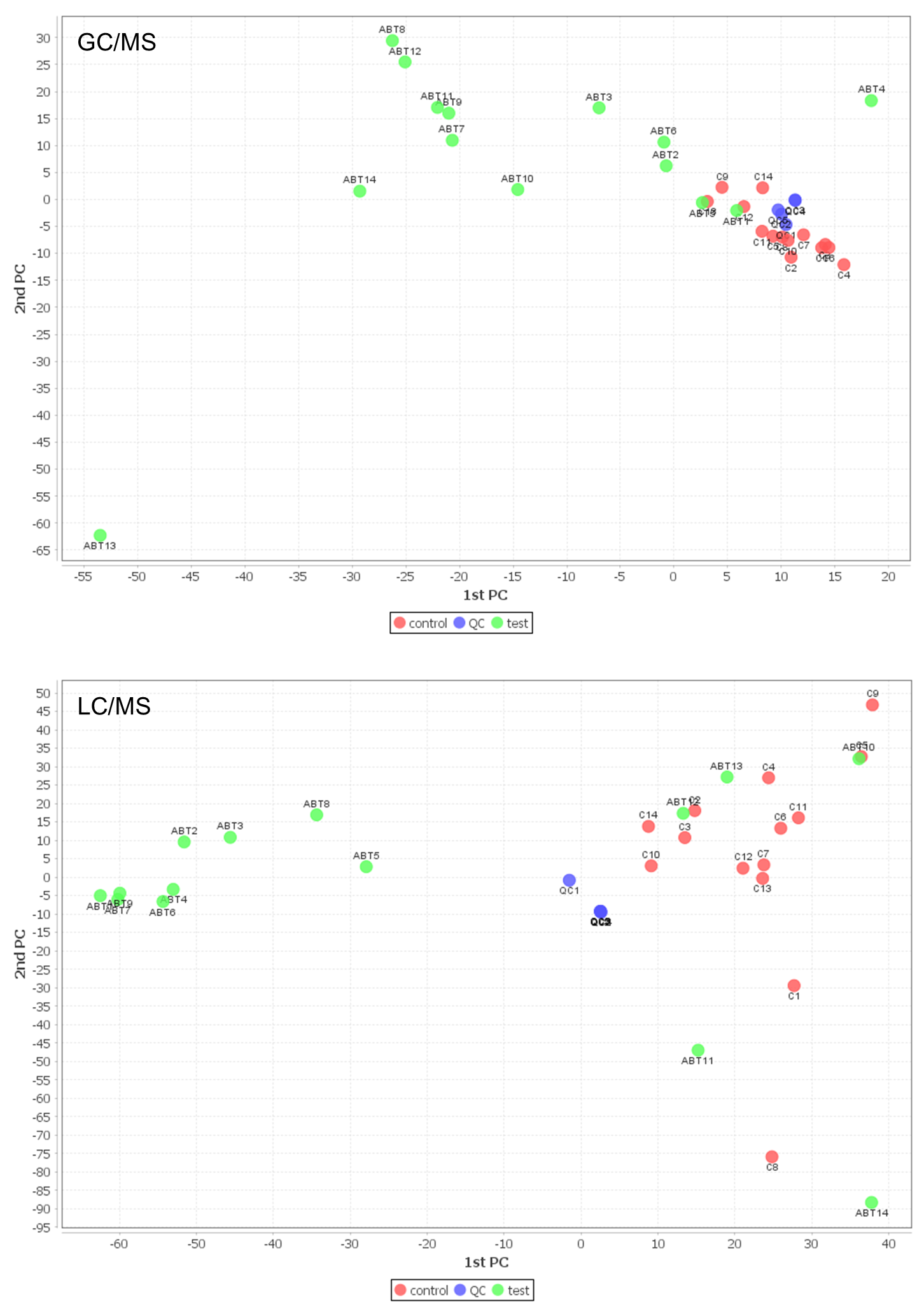
**

**Supplementary Figure 2.** **Box plots and kernel density plots before and after normalization.**

**
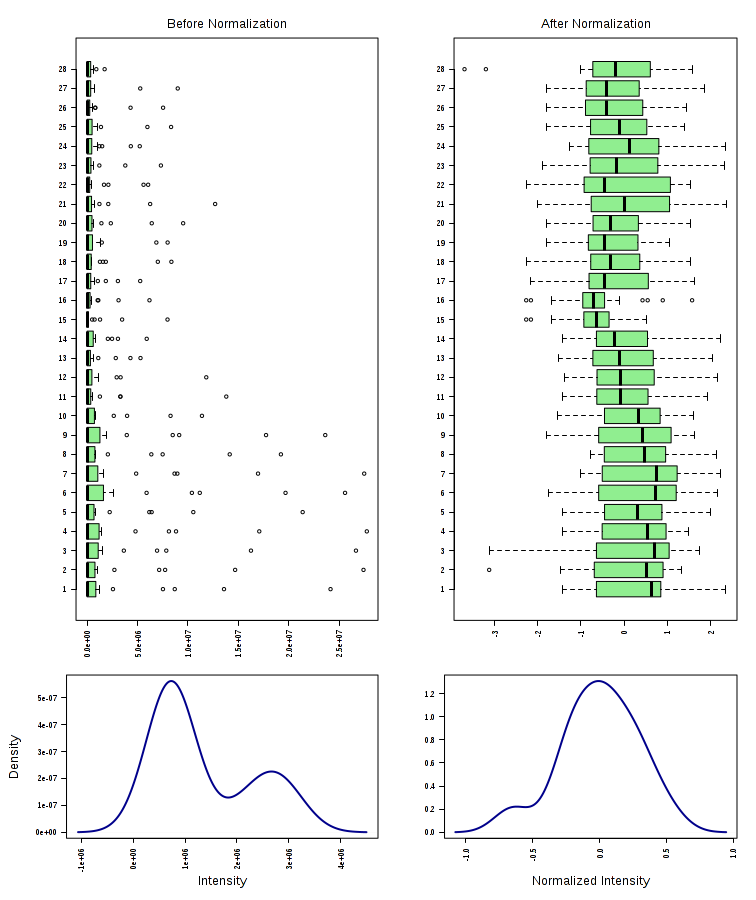
**

**Supplementary Figure 3. Box plots and kernel density plots before and after normalization.**


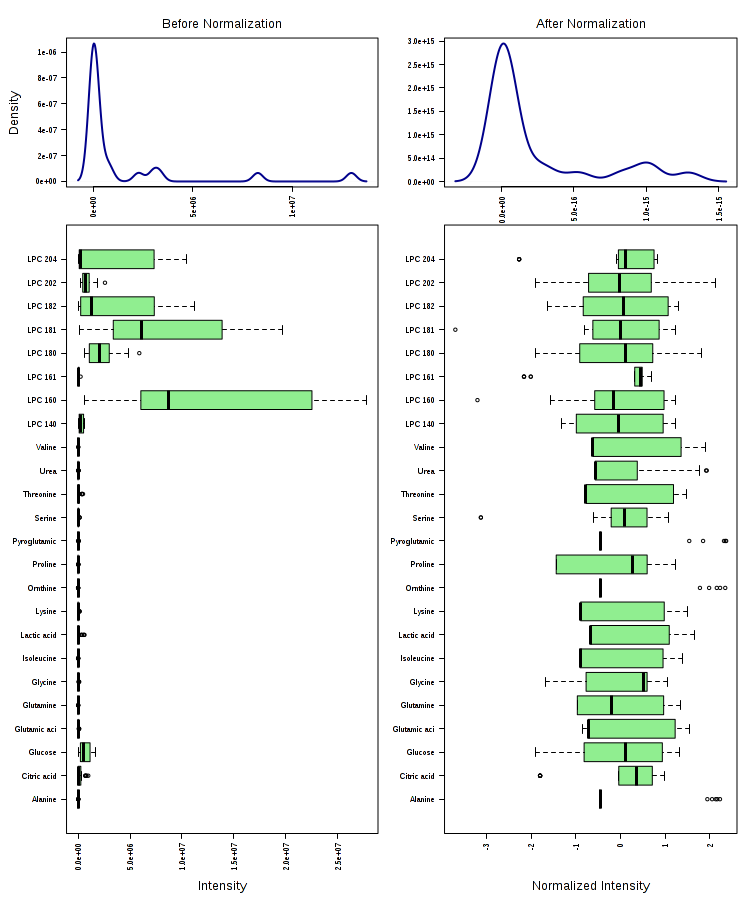


**Supplementary Figure 4. GC-MS spectrum of standards and chromatogram of QC sample.**

**
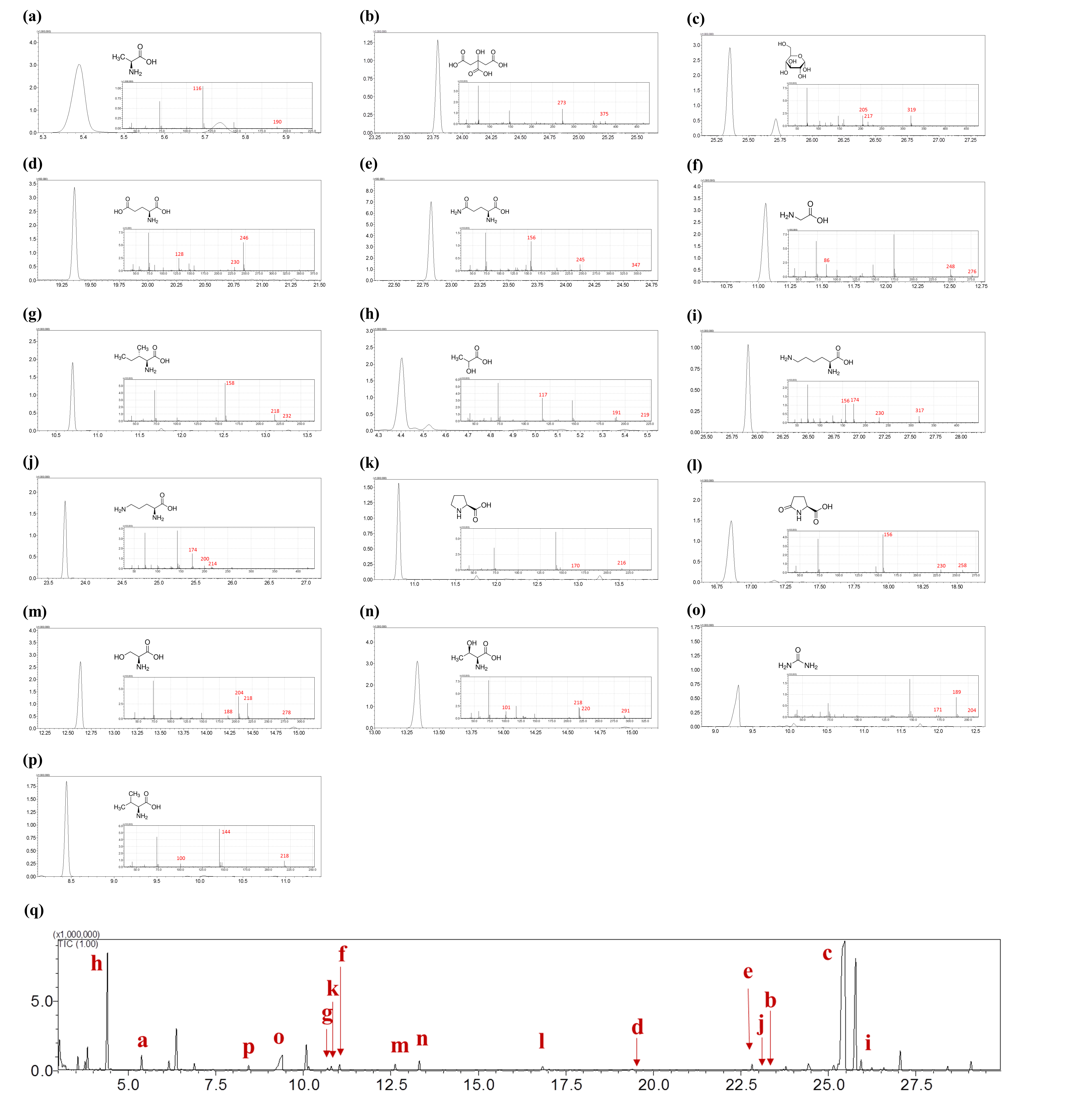
**

**Supplementary Figure 5. MS/MS spectrum of Lysophosphatidylcholine (17:0) standard.** (Collision energy: 10~50 eV)


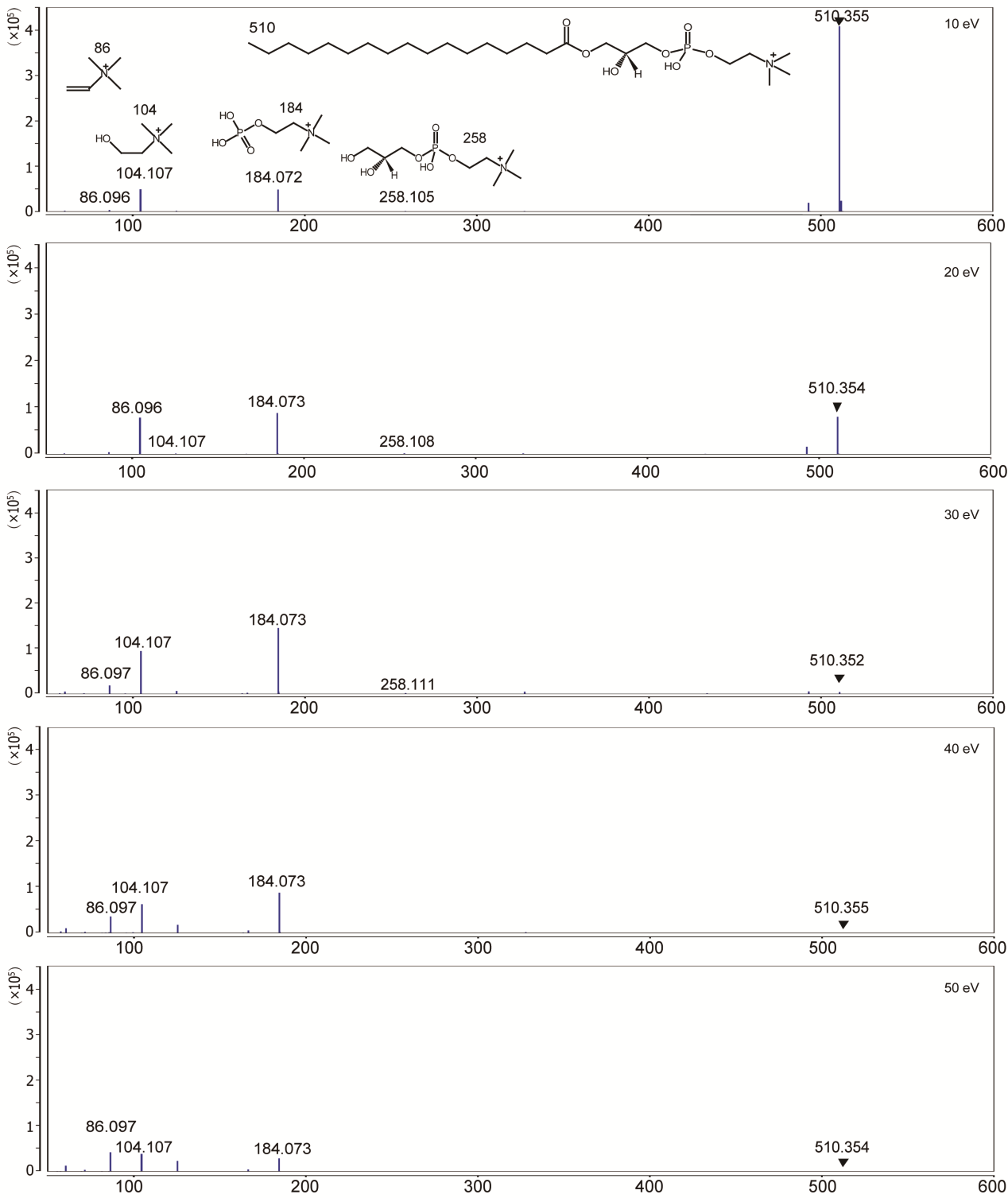


**Supplementary Figure 6. MS/MS spectrum of Lysophosphatidylcholine (14:0) which is an endogenous metabolite with significantly changed in allogeneic blood transfusion compared to autologous blood transfusion.** (Collision energy: 10~50 eV)


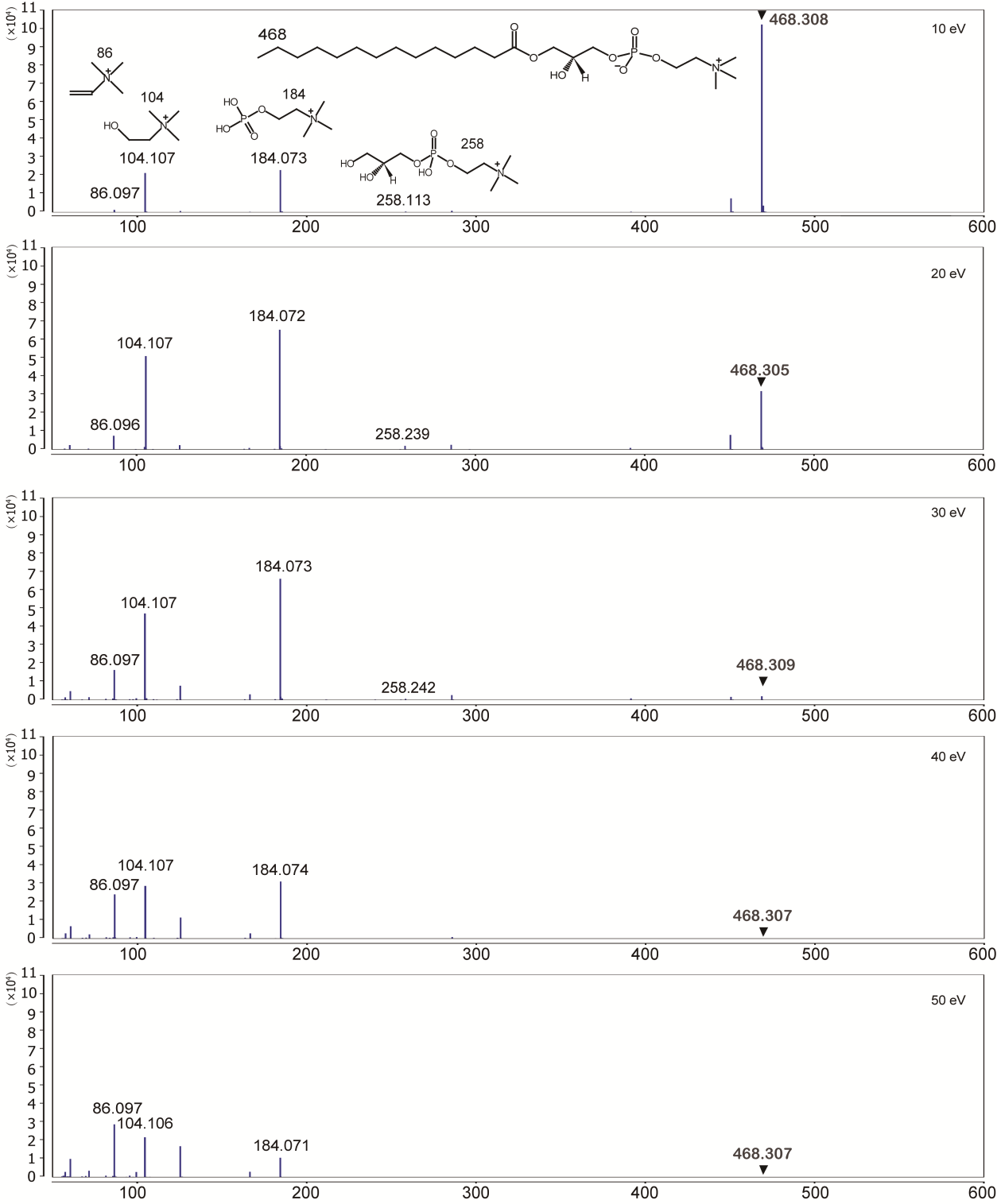


**Supplementary Figure 7. MS/MS spectrum of Lysophosphatidylcholine (16:0) which is an endogenous metabolite with significantly changed in allogeneic blood transfusion compared to autologous blood transfusion.** (Collision energy: 10~50 eV)


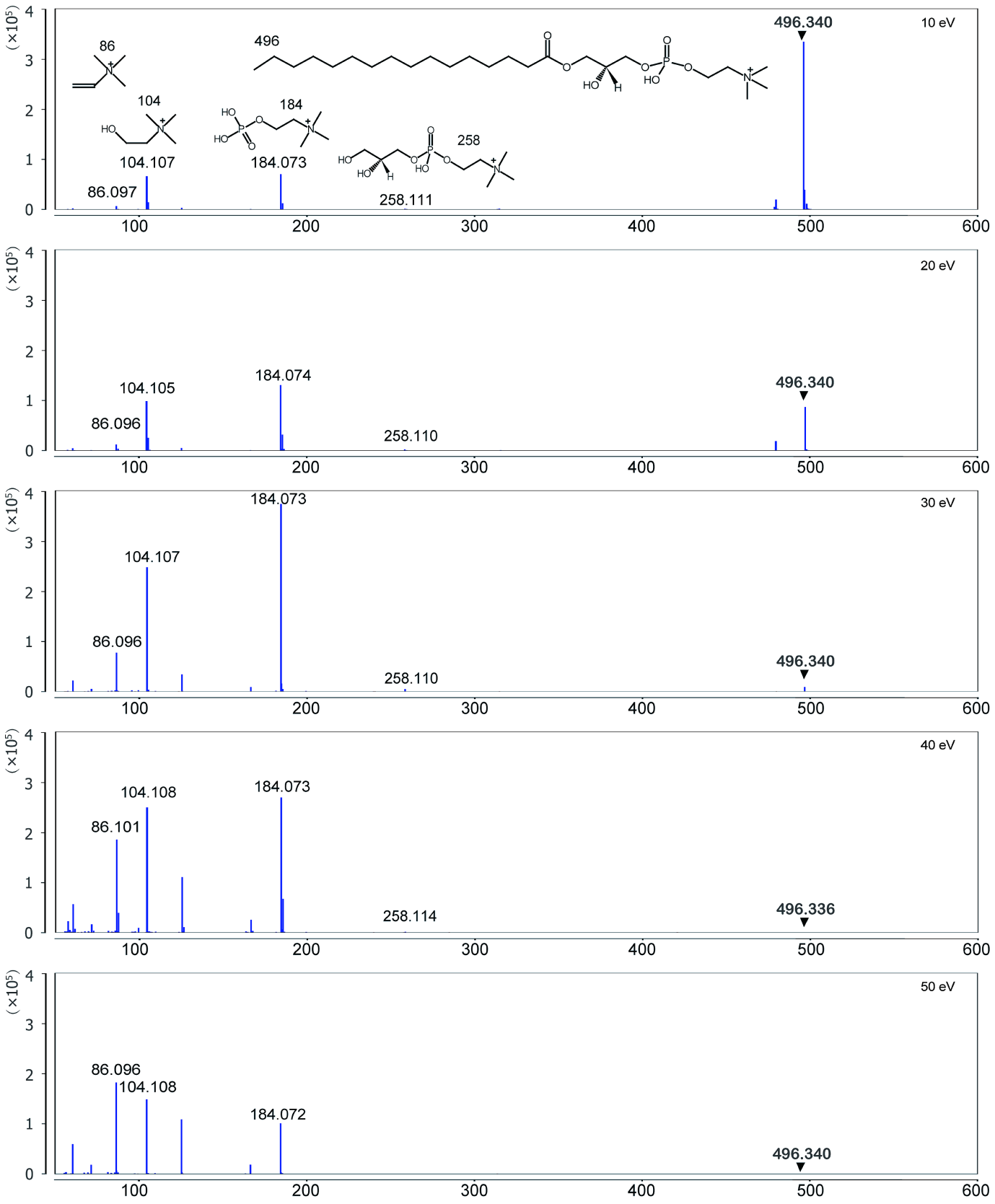


**Supplementary Figure 8. MS/MS spectrum of Lysophosphatidylcholine (16:1) which is an endogenous metabolite with significantly changed in allogeneic blood transfusion compared to autologous blood transfusion.** (Collision energy: 10~50 eV)


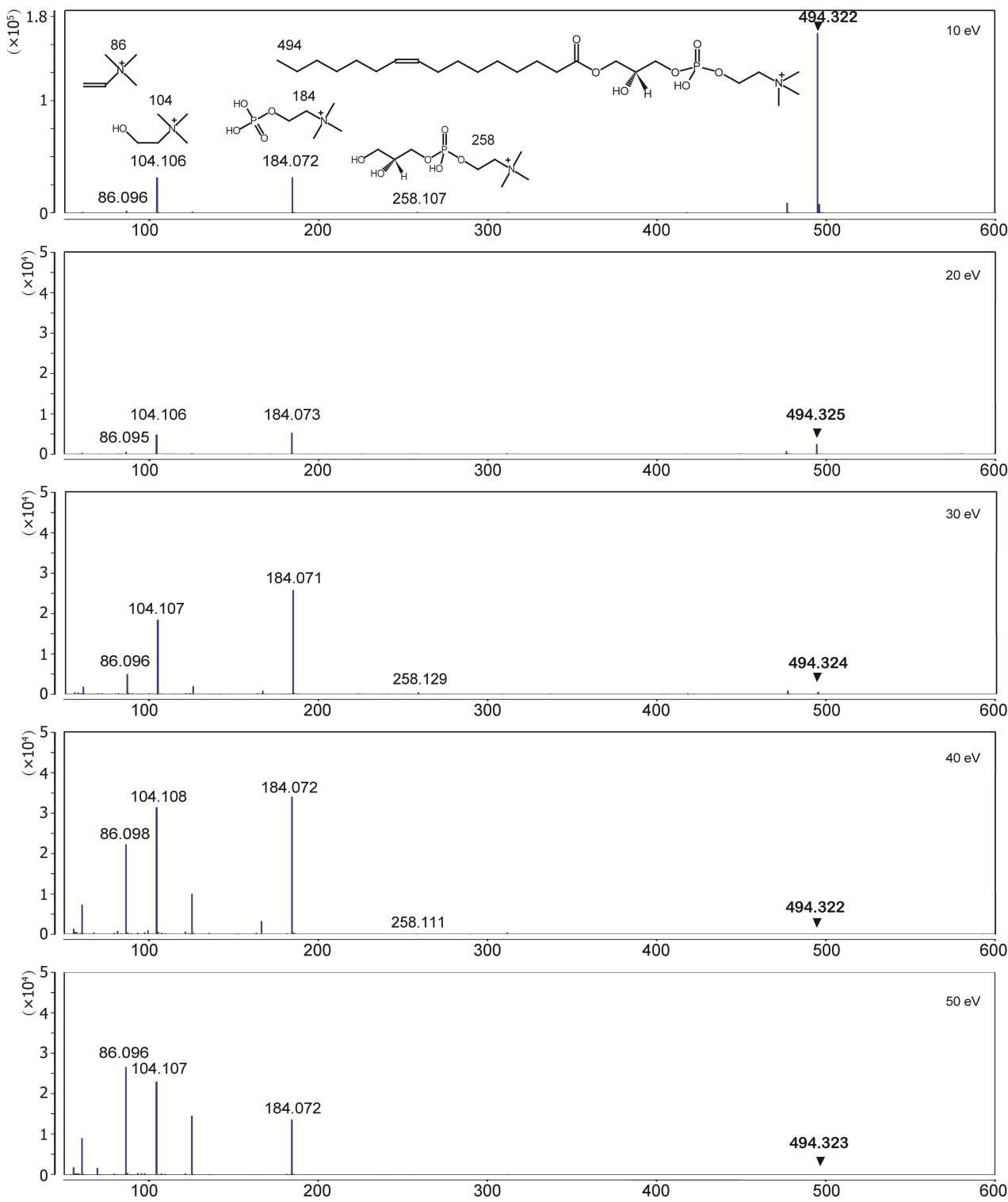


**Supplementary Figure 9. MS/MS spectrum of Lysophosphatidylcholine (18:0) which is an endogenous metabolite with significantly changed in allogeneic blood transfusion compared to autologous blood transfusion.** (Collision energy: 10~50 eV)


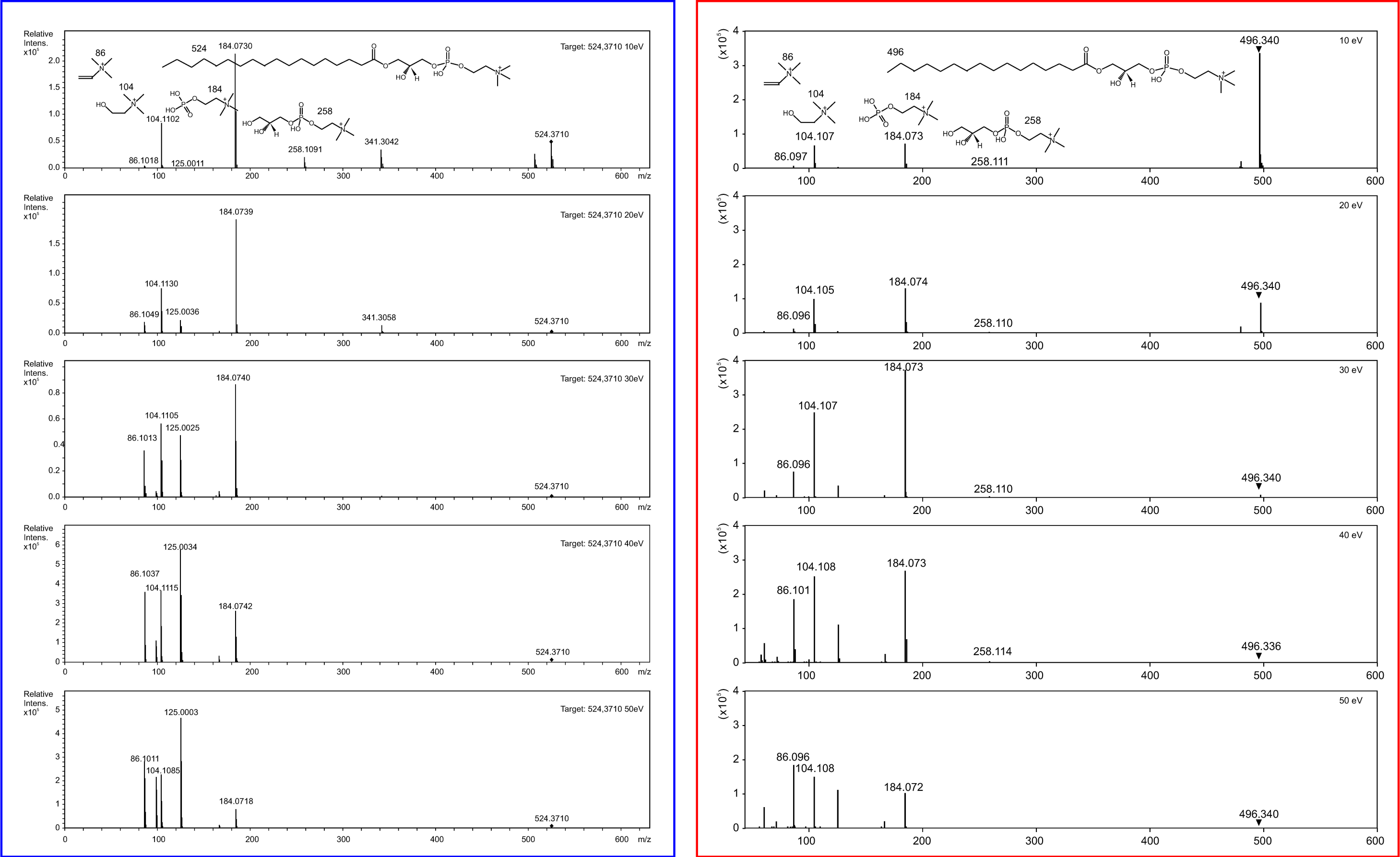


**Supplementary Figure 10. MS/MS spectrum of Lysophosphatidylcholine (18:1) which is an endogenous metabolite with significantly changed in allogeneic blood transfusion compared to autologous blood transfusion.** (Collision energy: 10~50 eV)


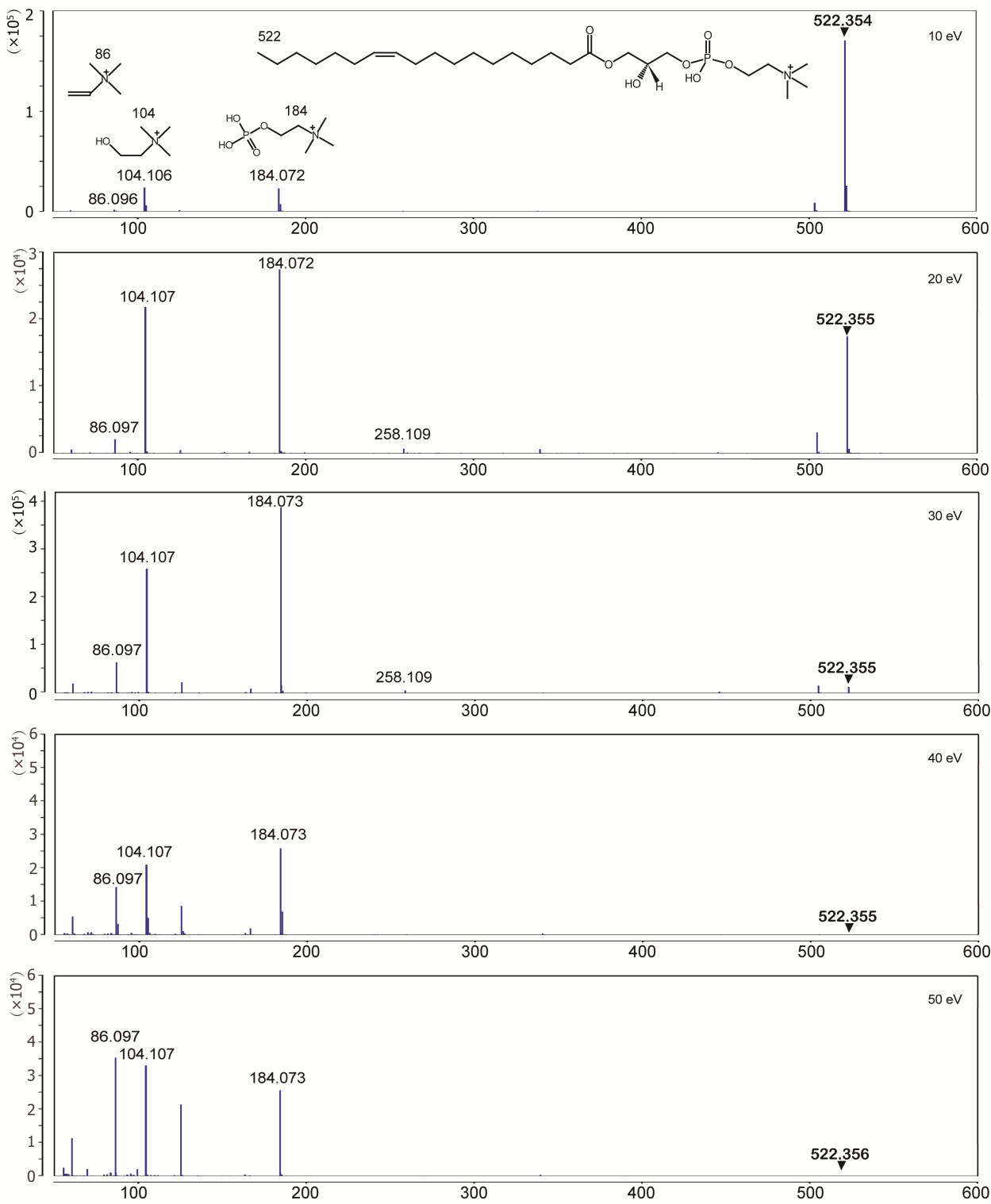


**Supplementary Figure 11. MS/MS spectrum of Lysophosphatidylcholine (18:2) which is an endogenous metabolite with significantly changed in allogeneic blood transfusion compared to autologous blood transfusion.** (Collision energy: 10~50 eV)


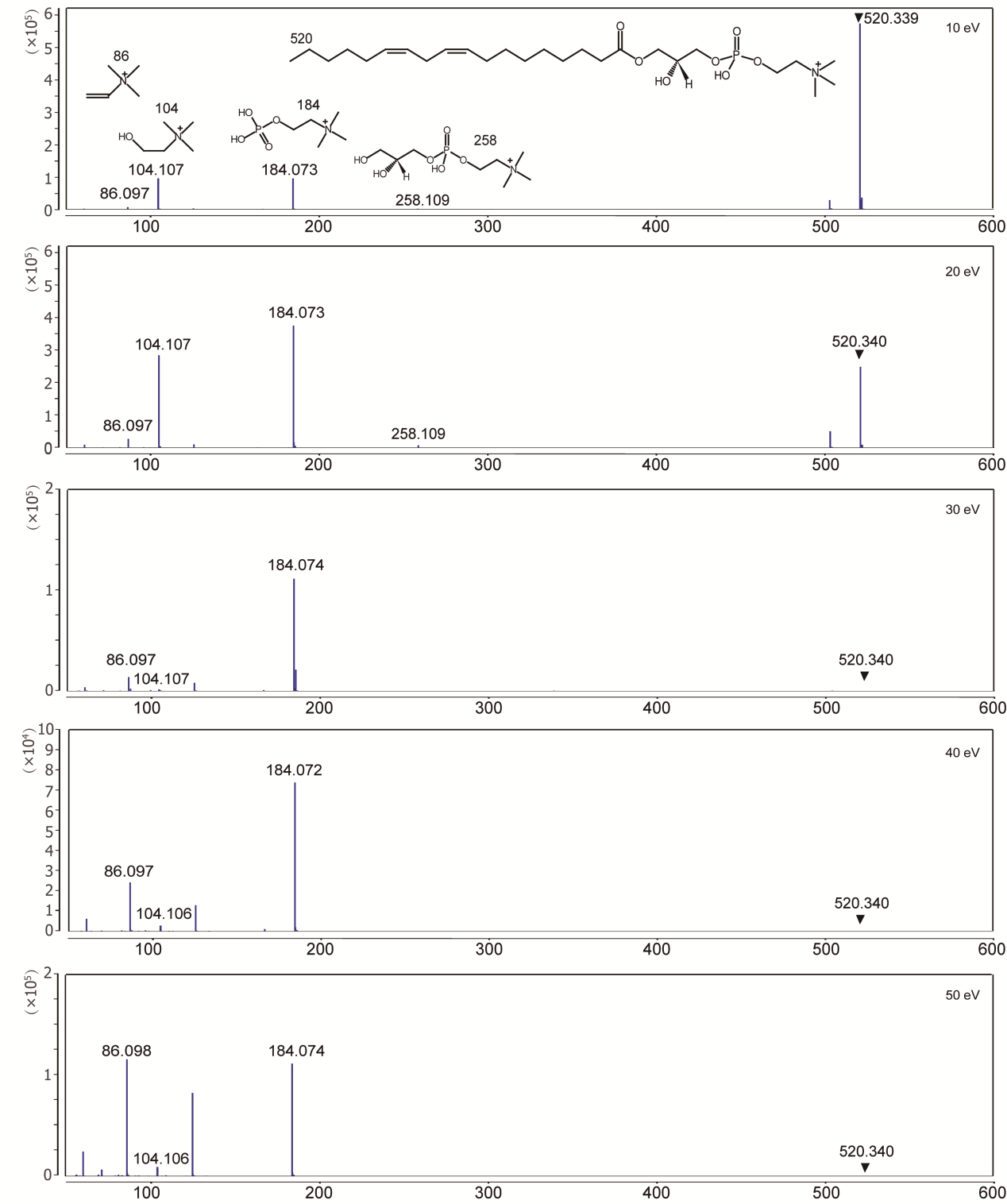


**Supplementary Figure 12. MS/MS spectrum of Lysophosphatidylcholine (20:2) which is an endogenous metabolite with significantly changed in allogeneic blood transfusion compared to autologous blood transfusion.** (Collision energy: 10~50 eV)

**
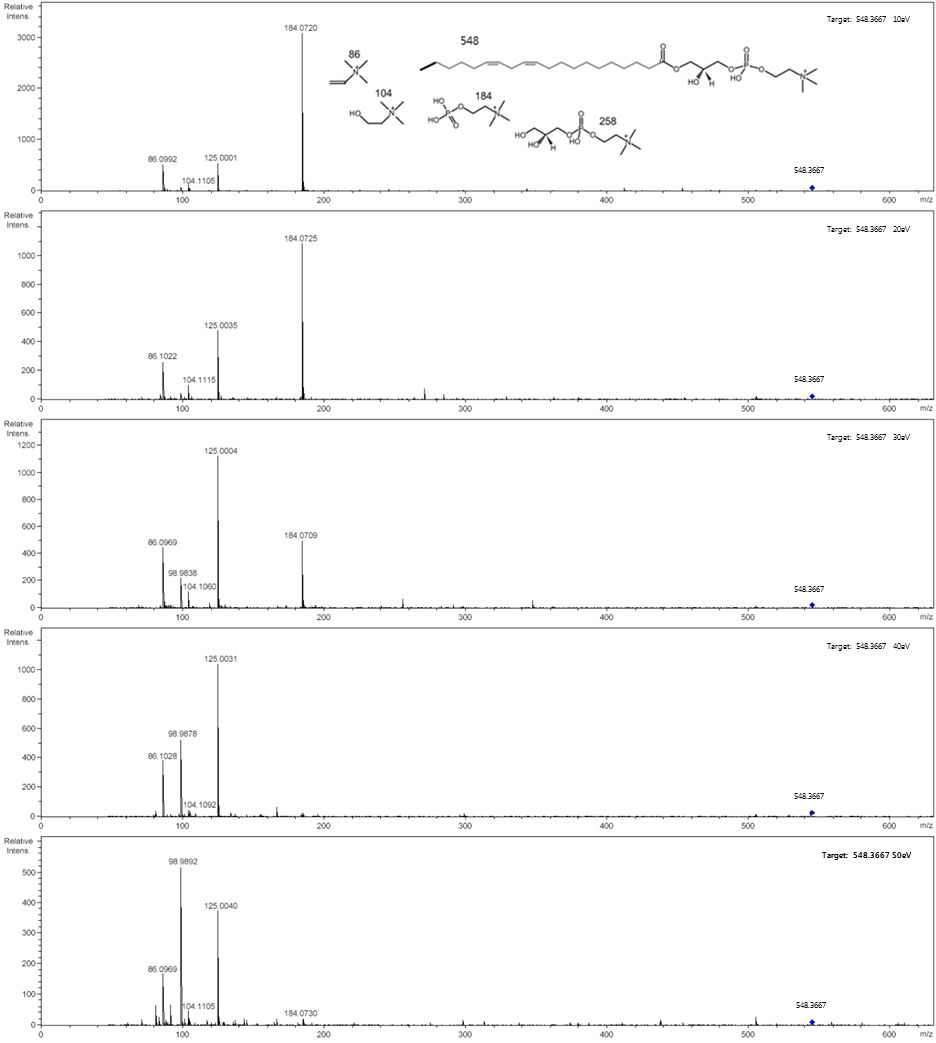
**

**Supplementary Figure 13. MS/MS spectrum of Lysophosphatidylcholine (20:4) which is an endogenous metabolite with significantly changed in allogeneic blood transfusion compared to autologous blood transfusion.** (Collision energy: 10~50 eV)


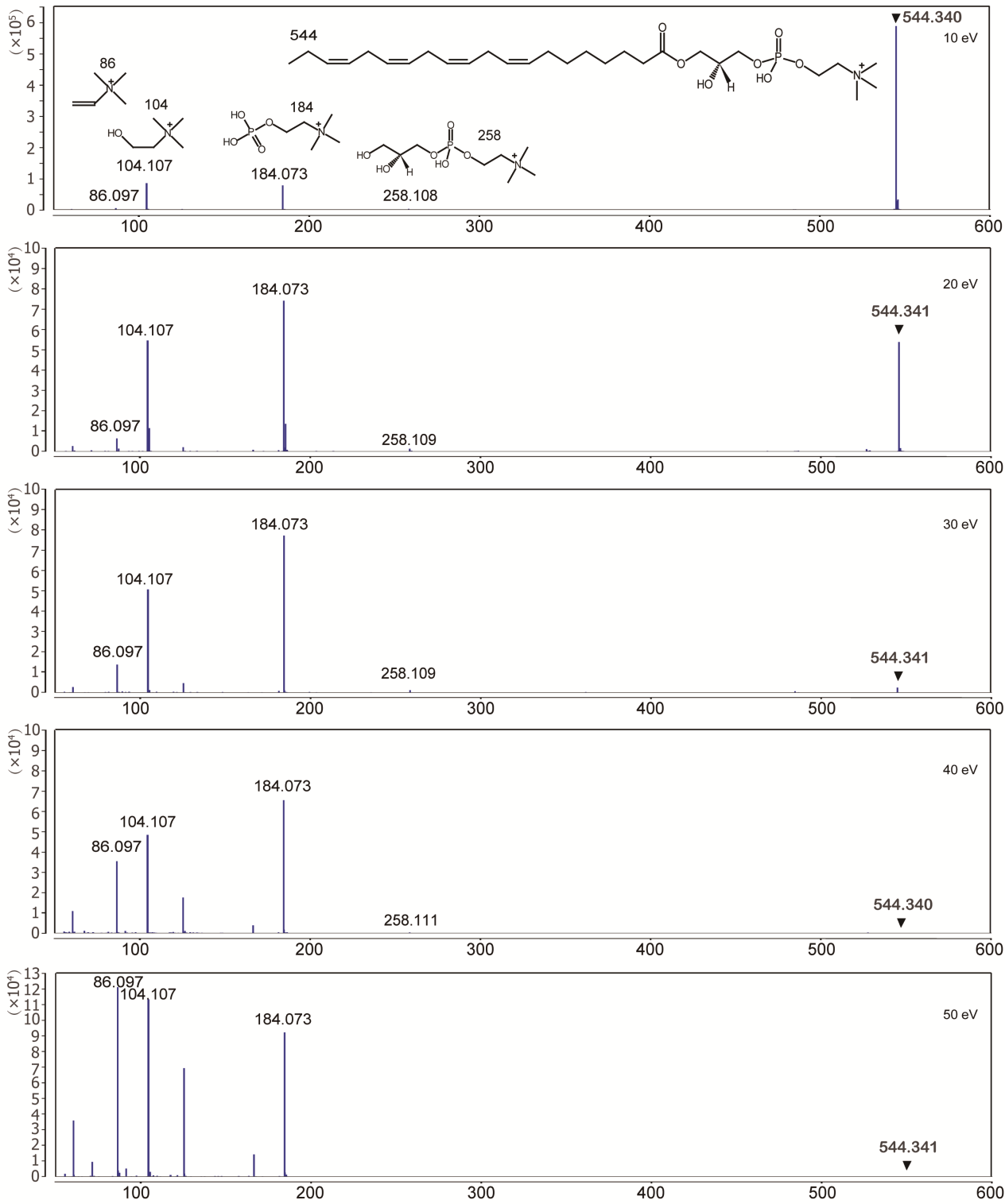


**Supplementary Figure 14. Results of Metabolite Set Enrichment Analysis (MSEA).**


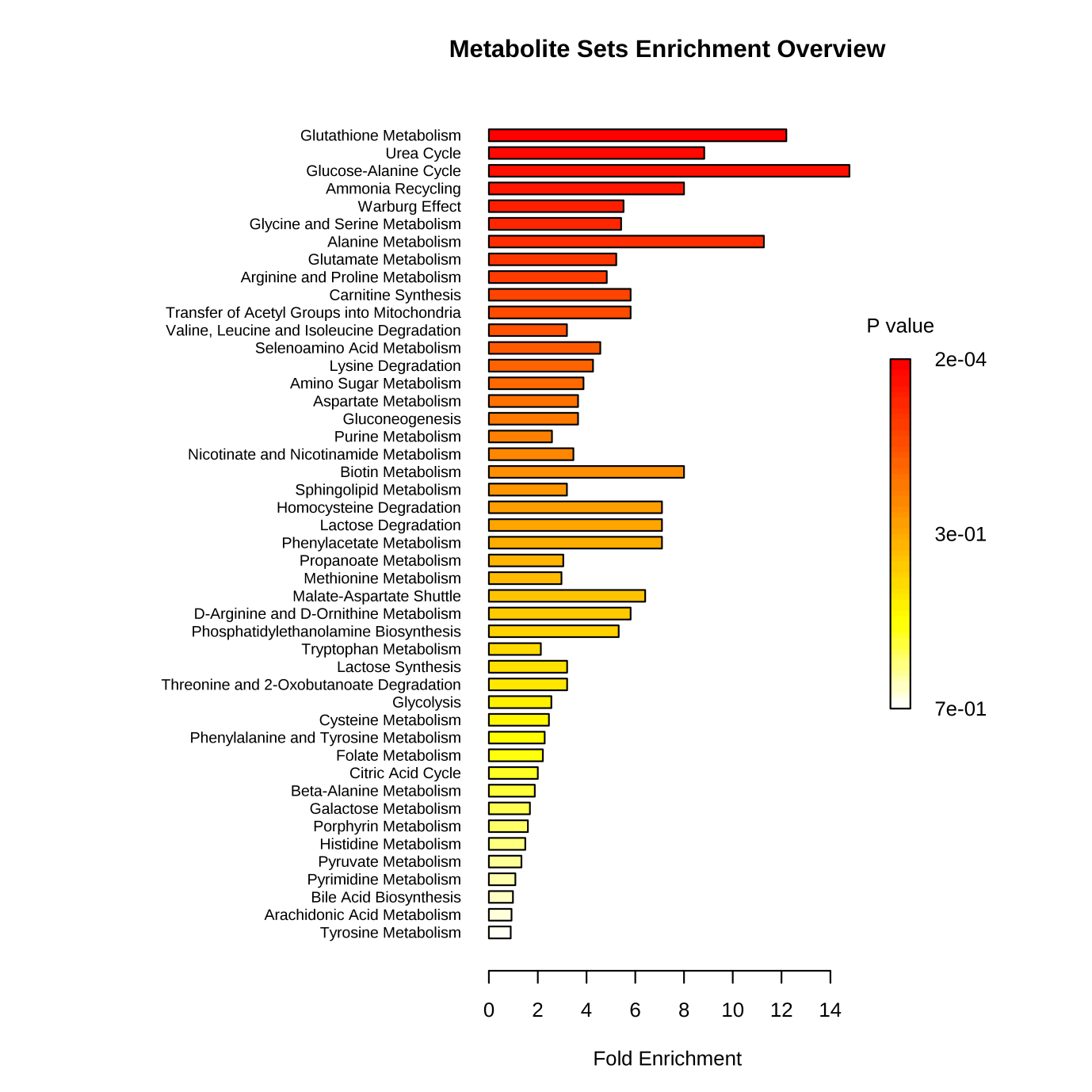


|  | total | expected | hits | Raw p | Holm p | FDR |
| --- | --- | --- | --- | --- | --- | --- |
| Glutathione Metabolism | 21 | 0.328 | 4 | 0.000204 | 0.0199 | 0.0199 |
| Urea Cycle | 29 | 0.453 | 4 | 0.000748 | 0.0726 | 0.0234 |
| Glucose-Alanine Cycle | 13 | 0.203 | 3 | 0.000815 | 0.0783 | 0.0234 |
| Ammonia Recycling | 32 | 0.5 | 4 | 0.0011 | 0.105 | 0.0234 |
| Warburg Effect | 58 | 0.906 | 5 | 0.00132 | 0.125 | 0.0234 |
| Glycine and Serine Metabolism | 59 | 0.922 | 5 | 0.00143 | 0.133 | 0.0234 |
| Alanine Metabolism | 17 | 0.266 | 3 | 0.00187 | 0.172 | 0.0261 |
| Glutamate Metabolism | 49 | 0.766 | 4 | 0.00551 | 0.502 | 0.0675 |
| Arginine and Proline Metabolism | 53 | 0.828 | 4 | 0.00733 | 0.66 | 0.0798 |
| Carnitine Synthesis | 22 | 0.344 | 2 | 0.0441 | 1 | 0.393 |
| Transfer of Acetyl Groups into Mitochondria | 22 | 0.344 | 2 | 0.0441 | 1 | 0.393 |
| Valine, Leucine and Isoleucine Degradation | 60 | 0.938 | 3 | 0.0621 | 1 | 0.507 |
| Selenoamino Acid Metabolism | 28 | 0.438 | 2 | 0.0683 | 1 | 0.515 |
| Lysine Degradation | 30 | 0.469 | 2 | 0.0772 | 1 | 0.531 |
| Amino Sugar Metabolism | 33 | 0.516 | 2 | 0.0912 | 1 | 0.531 |
| Aspartate Metabolism | 35 | 0.547 | 2 | 0.101 | 1 | 0.531 |
| Gluconeogenesis | 35 | 0.547 | 2 | 0.101 | 1 | 0.531 |
| Purine Metabolism | 74 | 1.16 | 3 | 0.103 | 1 | 0.531 |
| Nicotinate and Nicotinamide Metabolism | 37 | 0.578 | 2 | 0.111 | 1 | 0.531 |
| Biotin Metabolism | 8 | 0.125 | 1 | 0.119 | 1 | 0.531 |
| Sphingolipid Metabolism | 40 | 0.625 | 2 | 0.126 | 1 | 0.531 |
| Homocysteine Degradation | 9 | 0.141 | 1 | 0.133 | 1 | 0.531 |
| Lactose Degradation | 9 | 0.141 | 1 | 0.133 | 1 | 0.531 |
| Phenylacetate Metabolism | 9 | 0.141 | 1 | 0.133 | 1 | 0.531 |
| Propanoate Metabolism | 42 | 0.656 | 2 | 0.137 | 1 | 0.531 |
| Methionine Metabolism | 43 | 0.672 | 2 | 0.143 | 1 | 0.531 |
| Malate-Aspartate Shuttle | 10 | 0.156 | 1 | 0.146 | 1 | 0.531 |
| D-Arginine and D-Ornithine Metabolism | 11 | 0.172 | 1 | 0.16 | 1 | 0.559 |
| Phosphatidylethanolamine Biosynthesis | 12 | 0.188 | 1 | 0.173 | 1 | 0.585 |
| Tryptophan Metabolism | 60 | 0.938 | 2 | 0.24 | 1 | 0.784 |
| Lactose Synthesis | 20 | 0.312 | 1 | 0.272 | 1 | 0.834 |
| Threonine and 2-Oxobutanoate Degradation | 20 | 0.312 | 1 | 0.272 | 1 | 0.834 |
| Glycolysis | 25 | 0.391 | 1 | 0.329 | 1 | 0.976 |
| Cysteine Metabolism | 26 | 0.406 | 1 | 0.339 | 1 | 0.978 |
| Phenylalanine and Tyrosine Metabolism | 28 | 0.438 | 1 | 0.36 | 1 | 1 |
| Folate Metabolism | 29 | 0.453 | 1 | 0.371 | 1 | 1 |
| Citric Acid Cycle | 32 | 0.5 | 1 | 0.401 | 1 | 1 |
| Beta-Alanine Metabolism | 34 | 0.531 | 1 | 0.42 | 1 | 1 |
| Galactose Metabolism | 38 | 0.594 | 1 | 0.456 | 1 | 1 |
| Porphyrin Metabolism | 40 | 0.625 | 1 | 0.474 | 1 | 1 |
| Histidine Metabolism | 43 | 0.672 | 1 | 0.499 | 1 | 1 |
| Pyruvate Metabolism | 48 | 0.75 | 1 | 0.539 | 1 | 1 |
| Pyrimidine Metabolism | 59 | 0.922 | 1 | 0.616 | 1 | 1 |
| Bile Acid Biosynthesis | 65 | 1.02 | 1 | 0.653 | 1 | 1 |
| Arachidonic Acid Metabolism | 69 | 1.08 | 1 | 0.675 | 1 | 1 |
| Tyrosine Metabolism | 72 | 1.12 | 1 | 0.691 | 1 | 1 |
